# A Comparison of the Innate and Adaptive Immune Systems in Cartilaginous Fish, Ray-Finned Fish, and Lobe-Finned Fish

**DOI:** 10.3389/fimmu.2019.02292

**Published:** 2019-10-10

**Authors:** Nicole C. Smith, Matthew L. Rise, Sherri L. Christian

**Affiliations:** ^1^Department of Ocean Sciences, Memorial University of Newfoundland, St. John's, NL, Canada; ^2^Department of Biochemistry, Memorial University of Newfoundland, St. John's, NL, Canada

**Keywords:** innate immunity, adaptive immunity, chondrichthyes, actinopterygii, sarcopterygii

## Abstract

The immune system is composed of two subsystems—the innate immune system and the adaptive immune system. The innate immune system is the first to respond to pathogens and does not retain memory of previous responses. Innate immune responses are evolutionarily older than adaptive responses and elements of innate immunity can be found in all multicellular organisms. If a pathogen persists, the adaptive immune system will engage the pathogen with specificity and memory. Several components of the adaptive system including immunoglobulins (Igs), T cell receptors (TCR), and major histocompatibility complex (MHC), are assumed to have arisen in the first jawed vertebrates—the Gnathostomata. This review will discuss and compare components of both the innate and adaptive immune systems in Gnathostomes, particularly in Chondrichthyes (cartilaginous fish) and in Osteichthyes [bony fish: the Actinopterygii (ray-finned fish) and the Sarcopterygii (lobe-finned fish)]. While many elements of both the innate and adaptive immune systems are conserved within these species and with higher level vertebrates, some elements have marked differences. Components of the innate immune system covered here include physical barriers, such as the skin and gastrointestinal tract, cellular components, such as pattern recognition receptors and immune cells including macrophages and neutrophils, and humoral components, such as the complement system. Components of the adaptive system covered include the fundamental cells and molecules of adaptive immunity: B lymphocytes (B cells), T lymphocytes (T cells), immunoglobulins (Igs), and major histocompatibility complex (MHC). Comparative studies in fish such as those discussed here are essential for developing a comprehensive understanding of the evolution of the immune system.

## Introduction

The vertebrate immune system is divided into 2 subsystems—the innate immune system and the adaptive immune system. The innate immune system is the first to respond to initial infection and disease and does not retain memory of previous responses. Components of the innate immune system include physical barriers such as the skin, cellular processes such as phagocytosis and humoral components such as soluble proteins (1). If a pathogen persists, despite the innate immune defenses, the adaptive immune system is recruited. The adaptive immune system is highly specific to a particular antigen and can provide long-lasting immunity (2). While the innate immune system is assumed to have arisen >600 million years ago (MYA), specific components of the adaptive immune system, including immunoglobulins (Igs), T cell receptors (TCR), and major histocompatibility complex (MHC), are comparatively newer and are assumed to have arisen approximately 450 MYA in the first jawed vertebrates (i.e., Gnathostomata) (3–5). In order to understand the evolution and functionality of the immune system in jawed vertebrates, a comparative analysis of the key branches of Gnathostomata (Chondrichthyes, Actinopterygii, and Sarcopterygii) is required.

## Gnathostomata

Gnathostomes are subdivided into Chondrichthyes (cartilaginous fishes) and Osteichthyes (bony fishes). They diverged from a jawless common ancestor with the lineage leading to other bony vertebrates. While jawless fish have an adaptive immune system based on variable lymphocyte receptors (VLRs), B-like and T-like cells, Gnathostomes are the most distantly related group to mammals that have an adaptive immune system based on Igs, TCR, and MHC (3, 6).

**Figure 1:**
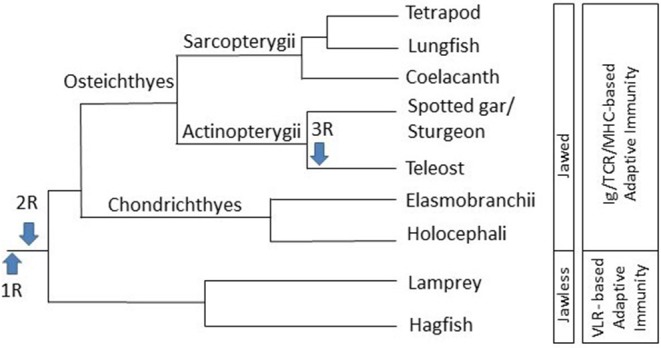
Schematic diagram of the evolution of jawed vertebrates and the immune system. Information sourced from multiple phylogenetic analyses (3, 4, 6, 9–11). R: genome duplication event.

There are over 1,000 species of cartilaginous fish, which are divided into two subclasses: Elasmobranchii (sharks, rays, skates, and sawfish) and Holocephali (chimeras) (7). The Osteichthyes are a diverse group of fish that have skeletons composed of calcified bone rather than cartilage and consist of over 40,000 species of fish (8). They are subdivided into two classes, the Actinopterygii (ray-finned fish) and the Sarcopterygii (lobe-finned fish) (Figure 1). The Actinopterygii have fins that are composed of webs of skin supported by bony spines, known as lepidotrichia. Ray-finned fish comprise 99% of the Osteichthyes, of which 96% are from the infraclass Teleostei (9, 12). Due to the large number of teleost species, as well their economic importance, there have been many genomic and functional immunological studies completed on teleost fish. The Sarcopterygii possess fleshy, lobed, paired fins, joined to the body by a single bone and are comprised of Actinistia (coelacanths) and Dipnoi (lungfish) (4). The majority of immunological studies on the cartilaginous fish and lobe-finned fish are genomic analyses, with very few functional studies. However, due to their unique position in the evolution of adaptive immunity, more functional studies are now being applied to cartilaginous fish. While there are several reviews that examine the innate or adaptive immune systems of Chondrichthyes and Actinopterygii, and some studies on Sarcopterygii (3, 13, 14), a comprehensive comparison of both the innate and adaptive immune systems in all 3 classes of fish is lacking. Thus here, we will endeavor to provide a comprehensive comparison of the innate and adaptive immune systems in cartilaginous fish, lobe-finned fish (focusing on coelacanths and lungfish), and ray-finned fish, with a focus on Teleost fish.

## The Innate Immune System

The innate immune system is the first to respond to an initial infection and/or disease. Elements of the innate immune response can be found in all multicellular organisms (14). The innate immune system can be categorized into three defense mechanisms: (1) physical barriers, (2) cellular components, and (3) humoral responses (15). As will be discussed, the functions of these defense mechanisms are highly conserved between fish and mammals.

## Physical Barriers

The first lines of defense in the fish innate immune system are physical barriers that prevent the entry of pathogens, which includes the skin (e.g., scales and mucus), gills, and epithelial layer of the gastrointestinal tract (15). One of the first physical barriers encountered by a pathogen is the skin. Fish are constantly immersed in an aquatic environment and as a result are continuously exposed to potential pathogens or other harmful agents. Therefore, the skin is extremely important in early prevention of pathogen invasion. Teleost skin has been shown to contain skin-associated lymphoid tissue (SALT) that consists of multiple cell types including secretory cells (e.g., goblet cells), lymphocytes (B and T cells), granulocytes, macrophages, and Langerhans-like cells (16, 17). In most teleost fish, the dermis layer of the skin consists of solid, bony scales known as leptoid scales. Interestingly, some teleost species, such as the catfish, have lost their scales during the course of evolution and instead some catfish species have regressed to having bony dermal plates covering their skin (18). The skin of cartilaginous fish also contains many cell types, including melanocytes, lymphocytes, macrophages, and granular leukocytes (19). The scales of cartilaginous fish are called placoid scales, also known as denticles (16). The skin of lobe-finned fish contains keratinocytes, granulocytes and B cells (20). Lobe-finned fish have cosmoid scales that includes a layer of dense, lamellar bone called isopedine. An equally important function of the skin is the ability to secrete mucus, which acts as both a physical barrier, by trapping pathogens, and a chemical barrier (16). Mucus from teleost fish contains a combination of lectins, lysozymes, complement proteins, and antimicrobial peptides (AMPs); all of which play a critical role in neutralizing pathogens (16, 21). While we hypothesize that skin mucus from both cartilaginous fish and lobe-finned fish contains these compounds as well, it has not been as extensively explored as in teleost fish. Supporting this hypothesis are studies showing that a transcript for a lectin, pentraxin, was found in the skin mucus of the common skate (*Raja kenojei*), while AMPs, including histones and S100 proteins, were found in the skin mucus of the African Lungfish (*Protopterus dolloi*) (20, 22).

In addition to being involved in osmotic balance and gas exchange, the gills are also an important physical barrier, having both innate and adaptive immune components. The physical barrier of the gills consists of the gill epithelium, a glycocalyx layer, and a mucus layer. In teleost fish, the interbranchial septum is reduced and contains a single caudal opening of the operculum, rather than multiple openings while in cartilaginous fish, the gills are supported for almost their entire length by an interbranchial septum with multiple branchial slits or gill openings (23). Immune cells, including macrophages, neutrophils and eosinophilic granulocytes have been observed in the gill associated lymphoid tissues (GIALT) of teleost fish (24). Lymphocytes have been identified in the gills of several teleost species (25, 26) and of the nurse shark (*Ginglymostoma cirratum*) (27). For example, B cells and T cells have been identified in the gills of rainbow trout (*Oncorhynchus mykiss*) and channel catfish (*Ictalurus punctatus*) while a specific B cell Ig transcript was observed in the gills of nurse shark (see adaptive immune section for a discussion on B cells, Ig, and T cells). Microbes present in the mucosal surface of the GIALT have been found to induce specific immunoglobulin producing B cells (28).

The gastrointestinal (GI) tract facilitates the absorption of nutrients, while preventing pathogen invasion through its epithelium. If a pathogen is ingested, it will encounter the GI tract, which, like the skin and gills, contains both innate and adaptive immune cellular components. Gut associated lymphoid tissue (GALT) can be found in both bony and cartilaginous fish; however, unlike in mammals, it is not highly organized but is composed of a diffuse network of myeloid and lymphoid cells. The intestine of teleost fish, especially the posterior segment, contains both innate and adaptive immune cells including macrophages, mast/eosinophilic granule cells, dendritic cells, B cells, and T cells (24, 29). Anal administration of *Vibrio anguillarum* to carp (*Cyprinus carpio*) and intraperitoneal injection of *V. anguillarum* to sea bass (*Dicentrarchus labrax*) resulted in the production of B-cells and Igs in the gut (30, 31). T cells have also been identified in the GALT of several teleost species (32–34). In teleost fish, as in mammals, the gut microbiota plays a major role in the development and maturation of the GALT, which in turn mediates its immune response (35, 36). For example, resident microbiota stimulates intestinal epithelial cell proliferation in the developing zebrafish intestine, while absence of microbiota prevents differentiation of the GI tract (37, 38). Dietary administration of probiotics to the gilthead seabream (*Sparus aurata*) enhanced the intestinal microbiota and increased expression of various immune genes in the intestine including MHCII and TNF-α, while administration of probiotics to the Nile Tilapia (*Oreochromis niloticus*) and rainbow trout promoted greater development of the intestine, as measured by villous height, and increased the population of intestinal granulocytes (39–41). Lymphoid aggregates, as well as macrophages and granular cells, have been found in the spiral valve of various shark and ray species (42, 43). Lymphocytes and macrophages appear in the gut of the Dogfish shark at hatching and their numbers increase with age, as determined by histological analysis (44). In addition, cytoplasmic Ig has been identified in some intraepithelial lymphoid cells of the shark gut and two Igs (one of high molecular weight and one of low molecular weight) were observed in the intestinal mucosa of the skate (*Raja kenojei*), although the exact Igs are unknown without the development of antibodies specific to detect cartilaginous fish Igs/proteins (42, 45). Large accumulations of lymphoid cells have been found in the gut of the Australian lungfish (*Neoceratodus forsteri*); however the cellular and molecular composition of these lymphoid masses is currently unknown (46). While there has been extensive research on the GALT of teleost fish, likely due to their economic importance, there are limited studies on the GALT of cartilaginous and lobe-finned fish and most are histological studies. It is unknown how the GALT in these species respond to infection and if it is in a similar manner as teleost fish and mammals. In addition, while the gut microbiome of some shark species has been identified (47), it is unknown how the microbiota effects the development of the GALT and its immune response in both cartilaginous fish and lobe-finned fish.

## Cellular Components

If a pathogen passes through the physical barriers, it will encounter the cellular and humoral aspects of the innate immune system. The cellular components of the fish innate immune system consist of many different cell types such as monocytes/macrophages, granulocytes such as mast/eosinophilic granule cells and neutrophils, dendritic cells, and natural killer cells. In bony fish, the primary sites for leukocyte production are the anterior (or head) kidney and thymus, while in cartilaginous fish, the primary sites include the epigonal organ, Leydig organ, thymus, and spleen (48). Analyses of possible sites of leukocyte production (such as the kidney and/or gonads) have yet to be studied in lobe-finned fish (49). Knowing the site of hematopoiesis in lobe-finned fish would allow for isolation of these cells and experiments that would lead to a better understanding of immune cells in these species.

When an innate immune cell encounters a pathogen, it will recognize a pathogen-associated molecular pattern (PAMP) found on the pathogen. Once recognized, the innate immune cell will become activated and can participate in several responses depending on their cell subtype including, but not limited to, phagocytosis and subsequent destruction of the pathogen, production of various cytokines and activation of the adaptive immune system via antigen presentation along with cytokine stimulation.

## Monocytes/Macrophages and Neutrophils

Monocytes/macrophages and neutrophils are the first to arrive and respond to initial infection. Macrophages are derived from hematopoietic progenitors which differentiate via circulating monocytes or via tissue resident macrophages. Differentiation of vertebrate macrophages is controlled by engagement of the colony-stimulating factor 1 receptor (CSF1R) (50). CSF1R has been characterized in several teleost species, and has been identified in the elephant shark (*Callorhinchus milii*) genome (51–54). Macrophages play a role in both the innate and adaptive immune systems and are key players during inflammation and pathogen infection, as well as in tissue homeostasis. In the innate immune system, macrophages of several teleost fish species have been demonstrated to destroy pathogens through phagocytosis, the production of reactive oxygen species (ROS) and nitric oxide (NO), and the release of several inflammatory cytokines and chemokines, similar to mammalian macrophages [reviewed in (55–57)]. In the adaptive immune system, macrophages are one type of professional antigen presenting cell (pAPC) that can present phagocytosed materials to the T lymphocytes of the adaptive immune system through a process termed antigen presentation. Macrophages in cartilaginous fish have not been studied as in depth as in teleost fish, however, it is known that nurse shark macrophages exhibit spontaneous cytotoxicity, a nonphagocytic killing mechanism (58). Lungfish macrophages are described to have typical vertebrate macrophage morphology (59, 60). Very few functional studies have been completed in lungfish, however, one study found that injection of lipopolysaccharide (LPS) did not change the number of macrophages in the coelomic cavity, as was expected (59). Similar to mammals, functionally distinct subpopulations of macrophages exist in bony fish. M1 (classically activated macrophages) are characterized by production of pro-inflammatory cytokines such as TNFα and IL-1β and production of ROS and NO, whereas M2 (alternatively activated macrophages) are linked to immunosuppression, wound repair and increased levels of arginase and anti-inflammatory cytokines such as interleukin (IL)-10 (55, 57, 61). The best characterized macrophage phenotype in teleost fish is comparable to M1 macrophages where they can destroy pathogens via acidification, nutrient restriction, production of reactive intermediates and various cytokines and chemokines (55–57). Macrophages, as well as virtually all immune cells, are able to communicate with each other via cell-derived extracellular vesicles (EVs) which contain and deliver messenger RNA (mRNAs), microRNA (miRNAs) and proteins (62, 63). While in recent years, EVs have been extensively studied in mammals, very few studies exist in fish. In one fish study, Atlantic salmon (*Salmo salar*) head kidney leukocytes were stimulated with CpG oligonucleotides which caused the release of EVs that contained mRNA and miRNA, as well as a protein composition similar to mammals including MHC I and MHC II molecules (64). The secretion of EVs was not induced by CpG in a splenocyte culture (containing mostly B cells) suggesting that the EVs were likely produced by macrophages or dendritic cells in the head kidney leukocyte culture (64). The existence of M1 and M2 cell populations, as well as EVs, have yet to be examined in cartilaginous and lobe-finned fish.

The most abundant granulocytes in bony fish are neutrophils, and like macrophages, neutrophils are critical to the innate defense against pathogens (65). Neutrophils exhibit potent antimicrobial responses through various intracellular and extracellular mechanisms including the release of granules containing cytotoxic and antimicrobial enzymes, the release of neutrophil extracellular traps (NETs), phagocytosis and the production of ROS and NO [reviewed in (57, 65)]. Some bony fish granulocytes have a similar appearance to that of mammalian cells (neutrophils) or avian cells (heterophils). Fish granulocytes exhibit a wide variation in morphology, numbers and types of cells between species causing much confusion regarding their nomenclature. For example, a study by Tavares-Dias et al. (66) identified only one type of neutrophil in channel catfish, while a study by Cannon et al. (67) reported heterophils instead of neutrophils. Granulocytes in cartilaginous fish are classified in three types based on size, shape, and staining properties. G1 granulocytes, referred to as heterophils or fine eosinophilic granulocytes, are often the most common granulocyte in cartilaginous fish. Their numbers can range from 20 to 50% of the total leukocytes in the blood, depending on species (68). G2 granulocytes resemble mammalian neutrophils, while G3 are referred to as coarse eosinophilic granulocytes (68, 69). G3 is more commonly seen in cartilaginous fish, compared to bony fish (68). Not all species of cartilaginous fish exhibit all three types of granulocytes; for example, only G1 and G3 granulocytes have been found in Thornback rays (*Raya clavate*) and small eyed rays (*Raja microcellata*) (68). In the African lungfish (*P. dolloi*), two types of granulocytes were identified in the South American lungfish (*Lepidosiren paradoxa*), three granulocyte types were identified based on Giemsa-staining and granule size (eosinophilic I, eosinophilic II and basophilic type) (70) and in the Australian lungfish (*N. forsteri*) four types of granulocytes have been described (basophil, neutrophils, large eosinophils and small eosinophils) (71).

## Recognition of Non-Self

Initiation of the innate immune response begins when germline-encoded intracellular or extracellular pattern recognition receptors (PRRs) of an immune cell bind to a PAMP found on a pathogen, such as bacteria-derived LPS, viral RNA, bacterial DNA, or a danger-associated molecular pattern (DAMP) found on proteins or other biomolecules that are released from stressed cells or injured cells. All PRRs have a domain for recognizing the PAMP that is coupled to a domain that interacts with downstream signaling molecules (72). In mammals, PRRs can be classified into at least five major groups: Toll-like receptors (TLRs), retinoic acid inducible gene I (RIG-I)-like receptors (RLRs), C-type lectins (CLRs), the nucleotide-binding domain, leucine-rich repeat containing proteins (NLRs), and absent in melanoma (AIM)-like receptors (73). Many homologs of mammalian PRRs have been identified in fish.

TLRs were the first PRRs to be discovered in fish and therefore have been the most extensively studied. To date there have been 13 TLRs identified in mammals, whereas over 20 have been identified in different fish species (73–76). A comparison of the TLRs found in mammals, cartilaginous fish, ray-finned fish and lobe-finned fish, as well as their ligands (in mammals and when known in bony fish) can be found in Table 1. Some mammalian orthologs of TLRs have not been identified in fish, whereas some TLRs, including soluble TLR5 (sTLR5), TLR13, TLR14, and TLR18-28 are “fish-specific” (77). For example, a sTLR5 has been identified in bony fish, including rainbow trout, and Atlantic salmon, whereas no sTLR5 has been found in mammalian genomes (82, 90, 91). Interestingly, TLR5, as well as TLR1, TLR2, and TLR6, are missing from the Atlantic cod (*Gadus morhua*) genome (87, 88). Some bony fish, including the zebrafish (*Danio rerio*), the Dabry's sturgeon (*Acipenser dabryanus*) and the yellow catfish (*Pelteobagrus hydrophila*), possess TLR4-like genes, while TLR4 is absent in other bony fish species, as well as absent in coelacanths and cartilaginous fish (56, 85, 86). TLR4 in fish, however, does not possess the ability to recognize LPS as it does in mammals (56). TLR27 was first identified and thought to only be found in the coelacanth genome but has since been identified in the spotted gar (*Lepisosteus oculatus*) and elephant shark (78, 89). TLR2, TLR3, TLR6, and TLR9 have been identified in the gray bamboo shark (*Chiloscyllium griseum*) genome whereas no TLR6 or TLR10 homolog has been identified in teleost fish. In addition, a novel TLR with sequence similarity to TLR4 and TLR13 in mammals, and TLR21 in teleost fish, has been identified in the whale shark (*Rhincodon typus*) (80, 83).

**Table 1:** TLRs present in mammals, ray-finned fish, lobe-finned fish, and cartilaginous fish.

**TLR**	**Ligand**	**Cartilaginous fish**	**Ray-finned fish**	**Lobe-finned fish**	**Mammals**
TLR1 (54, 75, 77–79)	Lipopeptide/Peptidoglycan (m)	–	+	+	+
TLR2 (54, 75, 77, 78, 80, 81)	Lipopeptide/Peptidoglycan	+	+	+	+
TLR3 (54, 79, 80, 82–84)	dsRNA	+	+	+	+
TLR4 (75, 79, 81, 85)	LPS (m)	–	+^*^	–	+
sTLR5 (57, 75, 79, 82)	Flagellin	–	+	+	–
mTLR5 (75, 79)	Flagellin	–	+	+	+
TLR6 (77–79, 83)	dsRNA	+	–	–	+
TLR7 (54, 75, 77, 79, 83, 84, 86)	dsRNA	+	+	+	+
TLR8 (59, 75, 77, 79–81, 83)	dsRNA	+	+	–	+
TLR9 (59, 75, 77–79, 83)	CpG, IFN-γ	+	+	+	+
TLR10 (78)	ND	–	–	–	+
TLR11 (79)	Profilin (m)	–	–	–	+
TLR12 (74)	ND	–	–	–	+
TLR13 (75, 77, 78, 80)	Bacterial RNA	+	+	+	+
TLR14 (75, 77–79)	ND	–	+	+	–
TLR18 (75, 77, 78)	ND	–	+	–	–
TLR19 (75, 77, 79)	dsRNA	–	+	–	–
TLR20 (75, 79)	ND	–	+	–	–
TLR21 (75, 78–80)	CpG DNA	+	+	+	–
TLR22 (75, 77–79, 87, 88)	dsRNA/Bacterial PAMPs	–	+	+	–
TLR23 (75, 79)	ND	–	+	–	–
TLR24 (75)	ND	–	–	–	–
TLR25 (75, 77, 88)	ND	–	+	–	–
TLR26 (75)	ND	–	+	–	–
TLR27 (77, 78, 89)	LPS/poly (I:C)	+	+	+	–
TLR28 (77)	LPS/poly (I:C)	–	+	–	–

(m) Represents ligand known in mammals but not fish; (+) represents identified; (–) represents not identified; ND represents not determined;

* *Only found in zebrafish (Danio rerio) and Chinese rare minnow (Gobiocypris rarus). It is important to note that ligands may be fish species specific*.

Due to genome duplication events, several paralogs of various TLRs exist in fish. Two rounds of genome duplication (1R and 2R) are thought to have occurred early in vertebrate evolution, before the Cyclostome/Gnathostome divergence, ~500–800 MYA (Figure 1) (10, 11). Evidence, such as an increase in the number of Hox gene clusters, indicates that an additional genome duplication event (3R) occurred early in the teleost lineage, after it split from the lobe-finned lineage 325–350 MYA, while an additional round of genome duplication (4R) occurred in some fish species, including salmonids, thus leading to several paralogs of genes, including TLRs (79, 92). Paralogous TLR4 and TLR8 genes have been identified in zebrafish (*D. rerio*) (81, 93), TLR8 in rainbow trout (76) and TLR3 and TLR7 in common carp (*C. carpio*) (84), while multiple copies of TLR7, TLR8, TLR9, TLR22, and TLR25 have been identified in the Atlantic cod (87). The high number and large diversity of fish TLRs are likely derived from their distinct and diverse evolutionary history and environments that they occupy [reviewed in (77)].

In addition to TLRs, differences in several other PRRs between ray-finned, lobe-finned and cartilaginous fish have been noted. While AIM has not been identified in teleost or cartilaginous fish, two HIN200 domains, a PAMP-recognizing protein domain characteristic of AIM in mammals, were discovered in the coelacanth genome (78, 94). A group of unique NLRs possessing a C-terminal B30.2 domain has been identified in teleost fish, but is missing from the coelacanth genome (78). Additionally, novel immune-type receptors (NITRs) which have been studied extensively in ray-finned fishes are missing from the coelacanth genome (78). While all three RIG-I-like receptors have been characterized in teleost fish, only RIG-I and MDA5 have been identified in the elephant shark and coelacanth genomes (54, 78). However, as more high quality, well-assembled, and annotated genomes become available for additional cartilaginous and lobe-finned fish, additional NITRs may be identified. These differences indicate that not only is pathogen recognition quite diverse in fish, it can also be lineage-specific.

## Phagocytosis

Phagocytosis is one of the most ancient and universal tools of defense against foreign material. This mechanism of defense is observed even in unicellular eukaryotes, predating complex multicellular life (57, 95–98). Binding of a pathogen to a PRR triggers phagocytosis in cells termed phagocytes. These include macrophages, monocytes, neutrophils and dendritic cells and are found in both bony and cartilaginous fish (57, 95–98). Recently, the existence of B cells with phagocytic ability was discovered in various teleost fish species including rainbow trout, Atlantic salmon, and Atlantic cod (99, 100). It is unknown if cartilaginous fish and lobe-finned fish have phagocytic B cells. After engulfment, the phagosome, containing the pathogen, binds to a lysosome, forming a phagolysosome, where the pathogen is killed by various means including the production of ROS and NO (57). Studies in shark, skate, lungfish and teleost fish have demonstrated both ROS and NO production in various leukocytes (65, 101).

## Humoral Responses

Humoral responses are mediated by macromolecules produced by cells and released into the extracellular fluids following infection by a pathogen. Some of the most studied humoral components in fish include the complement system, lysozyme, antimicrobial peptides, and acute phase proteins. These components have many different functions including the promotion of inflammation and phagocytosis and direct bactericidal effects.

## Complement System

**Figure 2:**
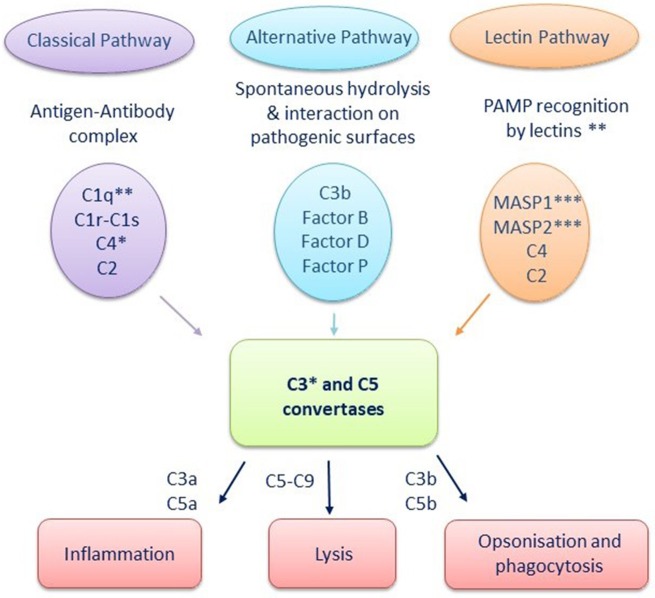
The three complement pathways with associated proteins. *Multiple C3 and/or C4 isoforms in some teleost and cartilaginous fish species. **Absence of mannose-binding lectin (MBL), ficolin, C1qA, and C1qC from genomes of any cartilaginous fish studied to date ***MASP2 transcript with no serine protease domain in hammerhead shark genome; MASP2 missing from elephant shark, little skate, and catshark genomes (102–109).

The complement system is a cascade of serum proteins that act cooperatively to mediate defense mechanisms including the elimination of pathogens through opsonization and phagocytosis and the promotion of the inflammatory response. The mammalian complement system is composed of ~30 proteins that make up three activation pathways: the classical pathway, activated by antibody-antigen complexes and thus a bridge between innate and adaptive immunity; the alternative pathway, which is independent of antibodies and activated directly by pathogens; and the lectin pathway which is activated by the binding of the mannose-binding lectin (MBL), or ficolin, to mannose (or other sugar) residues present on the pathogen surface (102). Figure 2 illustrates these three pathways, along with some of the associated proteins. Ultimately, these pathways induce activation of the C3 convertase, which cleaves inactive C3 into C3a, an anaphylatoxin that acts as a chemotactic factor and aids in inflammation, and C3b, which acts as an opsonin, as well as an activator of downstream complement proteins leading to the formation of the membrane attack complex (103, 104).

Most of the mammalian complement components have homologs in various teleost species, including rainbow trout (105), zebrafish (110), and channel catfish (111), among many others, and their functions have been well-characterized [reviewed in (56, 112)]. Similarly, components of all three pathways have been characterized in several cartilaginous fish species, where they have been found to have hemolytic properties (113–116). Furthermore, genes encoding complement components have been identified in lungfish (117, 118) and in the coelacanth genome (78). These studies in different fish classes/subclasses suggest that some components of the complement system are evolutionarily conserved and similar to those of higher vertebrates (102).

However, not all fish species contain all three pathways. MBL and ficolin genes have not been identified in any cartilaginous fish studied to date, while MASP2 transcripts are lacking in the elephant shark, little skate (*Leucoraja erinacea*) and catshark (*Scyliorhinus canicular*) (54, 106, 107). In addition, the hammerhead shark contains a MASP2 transcript that contains no serine protease domain, which is necessary to initiate the lectin pathway. This data suggests that the lectin pathway may not be present in cartilaginous fish (106).

Furthermore, some fish species contain multiple forms of various complement factors. Multiple C3 forms have been identified in teleost fish and cartilaginous fish. For example, rainbow trout have three C3 forms, common carp have eight, and gilthead seabream (*S. aurata*) have five (108, 109, 119), with each form demonstrating different binding efficiencies and functions. Two C3 variants have been described in the nurse shark and the small-spotted catshark, while two C4 gene haven been identified in the elephant shark and hammerhead shark (54, 106, 107). This structural and functional diversity suggests that these fish may have an increased capacity to recognize and destroy a broader range of pathogens compared to those with fewer forms, although this remains to be demonstrated.

## Lysozyme

Lysozyme is a lytic enzyme that acts on the peptidoglycan layer of bacterial cell walls by hydrolyzing 1–4 β-linked glycoside bonds resulting in lysis of the bacterium. It is also involved in other defenses such as opsonization and phagocytosis and activation of the complement system (120–122). Two types of lysozyme have been described in vertebrates: chicken (c)-type and goose (g)-type.

Lysozyme is one of the most studied innate immune components in fish. C-type and g-type lysozymes have been reported in several teleost species where they are found in neutrophils, monocytes and to a lesser extent in macrophages of several tissues (e.g., liver, kidney, spleen, gills) and in mucus (120, 123, 124). Recombinant (r-) c-type and g-type lysozymes have been found to have high bacteriolytic activity against a variety of pathogens of teleost fish such as *V. anguillarum, Aeromonas hydrophila*, and *Micrococcus lysodeikticus* (125, 126). A sequence homology search of the Atlantic cod genome revealed an absence of c-type lysozyme genes; however, four g-type lysozyme genes were identified in several different tissues (102). Intraperitoneal injection of *Francisella noatunensis*, an intracellular bacterium that commonly infects cod, stimulated the expression of two of the g-type lysozyme genes in the head kidney (127). The presence of multiple g-type lysozymes may compensate for the lack of c-type lysozymes in the Atlantic cod (127). The presence of lysozyme in the lymphomyeloid tissues of several cartilaginous fish was first discovered in 1979 (128). A genomic investigation by Venkatesh et al. failed to identify g-type lysozyme in the elephant shark genome, however c-type lysozyme was identified (54). This c-type lysozyme was characterized in the nurse shark and found to hydrolyze the cell wall of *M. lysodeikticus* and inhibit the growth of Gram-positive bacteria, suggesting a similar function for lysozyme as in teleost fish and higher vertebrates (129). In addition, two g-type lysozyme genes were discovered in the coelacanth genome, although no functional studies on lysozymes have been completed in coelacanth or lungfish to date (130). Collectively, these studies suggest that the function of lysozyme is similar in both bony and cartilaginous fish.

## Antimicrobial Peptides (AMPs)

AMPs, also known as host defense peptides, that are generally oligopeptides with a varying number of amino acids that are generally positively charged and play a major role in the innate immune system. AMPs protect against a variety of pathogens via disruptive or pore-forming actions against bacterial membranes. Over 90 fish AMPs have been identified and are characterized as β-defensins, cathelicidins, hepcidins, histone-derived peptides and fish-specific piscidins. Several of these AMPs have been cloned and subsequent functional studies have demonstrated antiviral and antibacterial activities against a variety of pathogens, demonstrating that AMPs from teleost fish exhibit many if not all of the characteristics of other vertebrate AMPs (131–134). For example, β-defensin has been characterized in gilthead seabream, where it demonstrated antimicrobial activity against *V. anguillarum*, while in Nile tilapia (*O. niloticus*) β-defensin has shown an inhibitory effect on the growth of *Escherichia coli* DH5α and *Streptococcus agalactiae* (135). Two cathelicidin genes have been identified in rainbow trout where they displayed activities against bacteria including *V. anguillarum* and *P. damselae* (136) while in Atlantic salmon, cathelicidin has demonstrated microbicidal properties against *V. anguillarum* (137). Unlike the comprehensive studies conducted on AMPs in teleost fish, research into shark and lobe-finned fish AMPs has not been as extensive. Two AMPs have been isolated from the dogfish shark (*Squalus acanthias*), transferrin (138) and squalamine (139), which were found to have potent bactericidal activity against both Gram-negative and Gram-positive bacteria. In addition, the AMP Kenojeninin I, has been isolated from the skin of fermented skate (*R. kenojei*) and was found to have inhibitory effects on *Bacillus subtilis, E. coli* and *Saccharomyces cerevisiae* (140). A recent study by Heimroth et al. (20) identified an increase in proteins with known antimicrobial function including histones and S100 proteins in skin mucus of the lungfish *P. dollo* during terrestrialization.

## Acute Phase Proteins

In both fish and mammals, tissue injury, infection and inflammation induce immune cells, such as macrophages, to secrete various cytokines into the bloodstream, which stimulate hepatocytes to produce and release acute phase proteins (APPs) (141, 142). APPs are classified based on the extent to which their concentrations change (minor, intermediate, or major) and the direction of change (positive or negative). They are involved in a variety of defense activities and include coagulation factors, such as fibrinogen and prothrombin, transport proteins such as ferritin, complement components, C-reactive protein (CRP) and serum amyloid proteins (SAP) [reviewed in (143)]. APPs are well-conserved in arthropods, fish, amphibians, and mammals (144). CRP and SAP are considered major APPs (e.g., their concentrations may increase up to 1,000-fold) and are the most extensively studied APPs in fish. They are members of the pentraxin family of APPs, are present in the body fluids of vertebrates and invertebrates, and are commonly associated with the acute phase response of inflammation (143). In addition to inflammation, CRP and SAP have been shown to activate the complement pathways and play a role in the clearance of apoptotic cells (143, 145).

Both CRP and SAP have been identified in several teleost species (146–148) where their levels in the serum have been shown to increase in response to various inflammation-inducing stimuli (149–152). For example, CRP and SAP expression in Atlantic salmon head kidney leukocytes are upregulated in response to r-IL-Iβ and r-IFNγ, two cytokines that stimulate APP production in mammals, suggesting that the acute phase response is evolutionarily conserved (151). Both CRP and SAP have also been identified in several different cartilaginous fish (153–155). CRP and SAP isolated from the serum of iridescent shark (*Pangasianodon hypophthalmus*) was found to agglutinate *Edwardsiella ictaluri* and *A. hydrophila* (156). Moreover, increased levels of CRP were found in the serum of sharks inhabiting a highly industrialized harbor estuary where exposure to polycyclic aromatic hydrocarbons (PAHs) and other contaminates was likely to lead to an inflammatory response (155). As well, transcriptome analysis of the Indonesian coelacanth, *Latimeria menadoensis*, genome identified SAP encoding transcripts (157), however, to our knowledge, no other studies examining CRP or SAP in coelacanths or lungfish have been reported.

## The Adaptive Immune System

If a pathogen persists, despite the innate immune defenses, the adaptive immune system will be activated. As previously stated, while jawless fish have an adaptive immune system based on VLRs, B-like and T-like cells, several components of the adaptive immune system, including Igs [also known as antibodies (Ab)], TCR and MHC, are believed to have arisen in the first jawed vertebrates (3, 6).

Like the innate immune system, the adaptive immune system includes both humoral and cellular components. B cells are key elements of the humoral adaptive immune response. The main role of B cells is to produce high affinity Ig against foreign antigen, and to act as a pAPC to present processed antigen to activate T cells. Abs occur in two forms: a soluble form that is secreted from the cell and a membrane-bound form that, in combination with the signaling molecules Igα/Igβ, forms the B cell receptor (BCR). T cells are key elements of cellular adaptive immunity. The T cell receptor (TCR) is always membrane bound and once stimulated via interaction with antigen presented by the pAPC, in the presence of co-stimulation, the T cell can be activated to function as a helper (CD4+) T cell, a regulatory (CD4+) T cell or a cytotoxic (CD8+) T cell.

**Figure 3:**
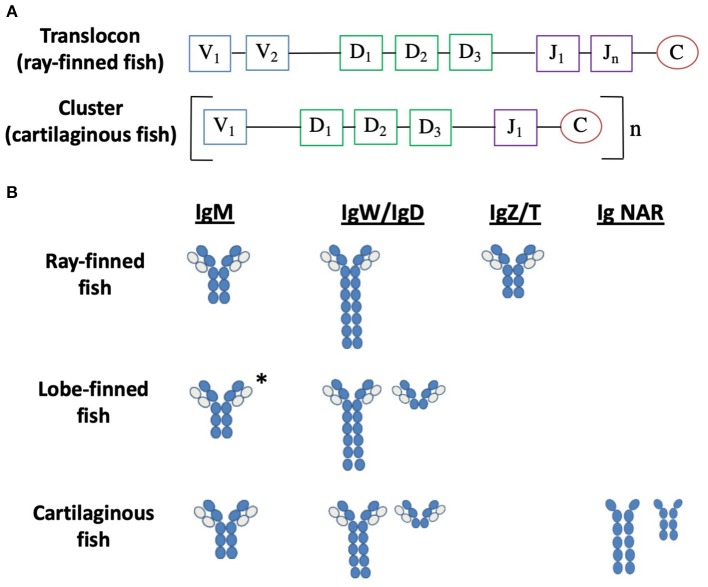
Antibody diversity and isotypes are divergent in fish. **(A)** Arrangement of the heavy chain loci in bony fish and cartilaginous fish. V represents variable segments, D represents diversity segments, J represents joining segments and C represents constant domains. **(B)** Examples of the immunoglobulin isotypes in fish. Dark blue circles represent heavy chain domains, light blue circles represent light chain domains (158–162). *IgM for lungfish only; no IgM in the coelacanth.

Antigen-specificity of B cells and T cells is determined by their BCR or TCR, respectively, which are formed from somatic recombination of variable (V), diversity (D) and joining (J) gene segments (Figure 3A), produced by the DNA-recombination ability of the RAG 1 and 2 enzymes and TdT (163, 164). RAG 1/2 and TdT enzymes, as well as the gene segments V, D, and J are present in all classes of jawed vertebrates [reviewed in (158, 165)]. This results in a highly diverse repertoire of BCRs and TCRs able to recognize innumerable different specific antigens and is unique to the adaptive immune system. Due to the random nature of the VDJ recombination, some BCRs and TCRs produced may recognize self-antigens as foreign. Therefore, developing B and T cells will undergo negative selection to ensure only cells that recognize foreign antigen survive. Negative selection occurs when a B cell recognizes self-antigen, inducing apoptosis or receptor editing, while positive selection occurs through antigen-independent signaling involving the BCR. In the case of T cells, a double positive T cell (CD4+ and CD8+) must bind MHC I or MHC II complex to be positively selected, which will induce the surviving T cell to become a CD8+ or CD4+ T cell, respectively. Negative selection occurs when a double positive T cells binds to MHC I or II with a high enough affinity to receive an apoptotic signal. While VDJ recombination has been characterized in fish [reviewed in (158, 165)], the process of negative and positive selection of developing B and T cells has not been fully elucidated, although these processes possibly occur in a similar manner as mammals. For example, double positive T cells were observed in the thymic cortex of sea bass, while single CD4+ or CD8 α+ cells were found in the thymic medulla, similar to that of mammals (166). There is little to no research on negative and positive selection of developing B and T cells in cartilaginous and lobe-finned fish, while there is very limited research in teleost fish. Studies into the regulation of autoimmunity would be valuable to better understand the mechanisms of negative selection in fish. The development of antibodies that specifically detect fish proteins, such as CD4 and CD8, is necessary to fully understand the homing and recirculation of B and T cells in fish.

## The Adaptive Humoral Response: B Cells

The BCR includes the membrane-bound antibody (Ab) and the Ig-α/Ig-β (CD79a/b) heterodimer, which is involved in signal transduction. Ab proteins are comprised of two heavy chains (IgH) and two light chains (IgL) held together by disulfide bonds forming a “Y” shaped quaternary structure (158). Both IgH and IgL chains contain one N-terminal variable domain (VH and VL) and one or more C-terminal constant domains (CH and CL). The arms of the “Y” are composed of one constant and one variable domain from each heavy and light chain and are the site of antigen binding, called the Fab region (fragment, antigen-binding). The base of the “Y” is composed of two heavy chain constant domains and is referred to as the Fc (fragment, crystallizable) region. The Fc region mediates the effector functions of the antibody by binding to a specific class of Fc receptors (and other molecules such as complement proteins) with the IgH categorizing them into specific isotypes. The variable regions of the heavy and light chain loci are assembled via somatic gene rearrangement from an array of multiple V, D, and J segments during B cell development, allowing each B cell to produce a unique Ab. In response to antigen, in combination with helper T cell interactions, B cells will secrete antigen–specific Abs. Three classes of Ab have been identified in both teleost fish and cartilaginous fish: IgM, IgD, and IgZ/T in teleost fish, and IgM, IgW, and IgNAR in cartilaginous fish, presumably each with different effector functions. In lungfish, IgM, IgW, and IgN have been identified, while in coelacanths two forms of IgW has been discovered (Figure 3B) [reviewed in (158)].

Similar to all vertebrates (except cartilaginous fish), the IgH genes of teleost fish are arranged in a translocon configuration of which multiple V segments are found upstream of several D and J segments, followed by C segments (Vn-Dn-Jn-C) (Figure 3A) (159). Depending on the species, differences may occur such as duplication of individual V, D, or J segments, or tandem duplication of C domain exons such as that found in Atlantic salmon and zebrafish (167, 168). Instead of the single translocon locus, the IgH loci of cartilaginous fish adopt a multiple mini-cluster organization, with each cluster consisting of one V, two or three Ds, and one J, followed by one set of C region exons for a specific isotype (Figure 3A) (169). The clusters can be repeated as many as 100 times in the genome, depending on the species. While most clusters are capable of rearrangement, some clusters are partially (VD-J) or fully recombined (VDJ or VJ) in the germline, a rearrangement that is unique to cartilaginous fish (170). IgH genes in lungfish are organized in a transiting form, having both cluster (like cartilaginous fish) and translocon (like teleost fish) configurations (160).

## IgM

IgM is the most ancient antibody class found in all jawed vertebrates; with the exception of coelacanths, which is the only known jawed vertebrate that does not contain IgM in the genome (171–173). IgM is the most prevalent Ab in both bony and cartilaginous fish plasma and can be found in both secreted and transmembrane forms. It shares a similar function in all jawed vertebrates, which includes mediating opsonization, antibody-dependent cell-mediated cytotoxicity, and complement activation, and thus contributes to both innate and adaptive immune responses (58, 102, 174, 175).

In teleost fish, IgM is multimerized into a tetrameric form, although there have been reports of a monomeric IgM form in some teleost species (176, 177). Due to an alternative splicing pathway, the transmembrane form of IgM is one domain shorter than the secreted form in teleost fish, resulting in a shortened IgM receptor on the B cell surface (178). The lack of this domain does not interfere with the ability to interact with Igα/Igβ signaling molecules (179). The J chain, which is required for IgM polymerization and secretion into the mucosa, has not been found in teleost fish, and therefore, tetrameric IgM is polymerized by interchain disulfide bonds (180). IgM is the only teleost isotype for which sub-isotypes have been identified. Two sub-isotypes of IgM have been identified in Atlantic salmon and brown trout (*Salmo trutta*), reflecting the pseudotetraploid state of salmonid genomes (181, 182).

In cartilaginous fish, IgM accounts for more than 50% of serum protein (158). Both the secreted and transmembrane forms of IgM contain four C domains, except in the neonatal nurse shark, where a subclass of IgM (IgM1_gj_) found in high amounts in the serum has only 3 C domains (169). IgM in the serum of cartilaginous fish is found in two different states, a monomeric 7S and pentameric 19S, which are present in approximately equal amounts (183). Pentameric IgM serves as the first line of defense, while 7S is produced later (183). Both 7S and 19S IgM play a role in cytotoxicity reactions via phagocytosis (58). In some cartilaginous fish species, such as the nurse shark, the J chain is present in pentameric IgM, although it may not be involved with IgM secretion, unlike the J chain in mammalian IgM (161, 184).

In contrast to the coelacanth, which does not contain IgM in the genome, lungfish species express multiple diverse IgM genes which vary among species (160, 185). For example, the West African lungfish has three IgM isotypes, while the spotted lungfish (*P. dolloi*) has two. Recently the J chain was identified in the spotted lungfish (186).

## IgD/IgW

IgD is found in many vertebrate classes, including teleost fish and acipenseriformes (a group of fish that phylogenetically links elasmobranches, teleosts, and sturgeons). It is orthologous to IgW (also known as IgX, IgNARC, or IgR depending on the species), which is found only in cartilaginous fish (187, 188), lungfish, and coelacanths (172, 173, 185), suggesting that IgD/IgW is as phylogenetically old as IgM (189, 190). The function of IgW and IgD, however, is poorly understood in both fish and mammals.

Teleost fish contain many forms of IgD, with constant domains ranging from 2 to 16 (191–193). IgD has only been found in a transmembrane form, with the exception of the channel catfish and the Japanese puffer (*Takifugu rubripes*), which contain both membrane and secretory forms (159). Teleost IgD is unique in that it is a hybrid of the CHμ1 domain followed by a varying number of CH-δ domains, depending on the species (194–197). The IgD heavy chain has not been identified in any teleost fish without the CHμ1 domain (195, 198, 199). IgD is co-expressed with IgM in most teleost fish, although they are absent in channel catfish and rainbow trout. Three different types of IgD+ cells have been identified in catfish: small IgM+/IgD+ B cells, larger IgM–/IgD+ B cells and granular cells containing exogenous IgD via a putative IgD-receptor. In rainbow trout, the ratio of IgD to IgM in the gills is much higher than other tissues. As well, an IgM–/IgD+ B cell subset has been found mainly expressed in the gills, indicating a role for IgD in the gills (191–193).

IgW in cartilaginous fish contains six to eight C domain exons, in addition to the V, D, and J segments. Multiple splice forms of IgW exist in cartilaginous fish other than the original six C domains (IgW-long), including a two C domain (IgW-short) form and a four C domain form (188, 200, 201). A V-less form of IgW has been identified in both the spiny dogfish (*S. acanthias*) and the nurse shark but represents only 8% of the IgW transcripts analyzed (200).

Two IgW transcripts have been identified in the African lungfish (160). Similar to cartilaginous fish, lungfish IgW can be found in a long form, consisting of seven C domains (homologous to IgW-long) or a short form, consisting of two C domains (160, 185). Two distinct loci for IgW have also been discovered in the Indonesian and African coelacanth (*Latimeria chalumnae*) (173). It remains unknown if the short and long forms of IgW found in cartilaginous fish and in lungfish have different effector functions and if the functions of IgD/IgW are species specific.

## Species Specific IgS: IgNAR, IgZ/T, IgQ

IgNAR (new/nurse shark antigen receptor) is a heavy-chain only Ig found only in sharks. Each chain of IgNAR contains a single-domain V region that can bind to antigen independently (202). IgNAR exists in both long and short forms, which can vary between species (183). The long transmembrane and secreted forms consist of five C domains while the short transmembrane form consists of three C domains (184, 203). Serum levels of IgNAR are much lower than IgM and it is unknown if the J chain is required for IgNAR multimer formation (8).

The immunoglobulin IgT/Z is produced only in bony fish and was first identified in rainbow trout (IgT) and zebrafish (IgZ) (167, 199). In most bony fish characterized to date, IgT/Z contain four C domains, although this is known to vary in a number of species (204–206). While only a few studies have been performed, it is thought that IgT is specialized for mucosal immunity and functions analogously to mammalian IgA. For example, the concentration of IgT/Z in the serum of rainbow trout is much lower than that of IgM, the ratio of IgT/Z:IgM is 63 times higher in the gut than in the serum (207). This study also demonstrated that, following intestinal parasitic infection, the number of IgT+ B cells increased in the gut, but the number of IgM+ B cells in the gut did not change (207). In addition, IgT+ B cells are also found in teleost skin associated lymphoid tissue (SALT) where they secrete IgT into skin mucus (17).

High-throughput sequencing of two species of African lungfish (*P. dolloi* and *P. annectens*), followed by Southern blot, identified two unique Ig isotypes in lungfish; these include 3 IgN isotypes (IgN1 found only in *P. dolloi* while IgN2 and IgN3 found only in *P. annectens*) and IgQ (found only in *P. annectens*) (160). Both IgN and IgQ are thought to originate from the IgW lineage (160).

## B Cell Response and Immunity

Both bony and cartilaginous fish lack bone marrow, the main site of hematopoiesis in mammals, and germinal centers (GC), specialized sites where mature B cells proliferate, differentiate, and selection of high affinity BCR occurs in mammals. Instead, in teleost fish, the main site of hematopoiesis is the anterior (or head) kidney. Progenitor B cells and plasma cells are found in the anterior kidney, while mature B cells and plasma blasts are found in the posterior kidney and in the spleen (208, 209). Evidence for B cell development in the anterior kidney is supported by expression of RAG-1/2 and terminal deoxynucleotidyl transferase (TdT), and the resulting development of immature B cells with membrane Ig on their surface. It is proposed that mature B cells are released into the blood where they encounter antigen and mature into plasma blasts or plasma cells. Plasma cells then migrate back to the anterior kidney where they may become long-lived plasma cells, supporting the storage of Ig-secreting cells (208, 210). However, more work is required to fully elucidate the mechanisms regulating homing of B cells in fish. The spleen is considered the only secondary lymphoid organ (SLO) in teleost fish, where expression of AID (see below) has been observed, suggesting that the spleen is the site for antigen stimulation (211).

In cartilaginous fish, the Leydig organ, a gland-like structure associated with the esophagus, and the epigonal organ, a structure physically attached to the gonads with a similar structure and organization as the Leydig organ, are the main sites of hematopoiesis and B cell production (48). Lymphocytes of various sizes are abundant in these organs and form a loose follicle-like aggregate with scattered plasma cells (212). While most cartilaginous species have both organs, some species only have one, such as the nurse shark, which only has an epigonal organ (48). Like bony fish, RAG1 and TdT expression in the epigonal organ provides evidence that it is a site of B cell development (213). Additionally, hematopoietic transcription factors important in B and T cell development are expressed in the Leydig and epigonal organ of the embryonic clearnose skate (*Raja eglanteria*) (214). The spleen of cartilaginous fish contains well-defined white pulp (WP) and red pulp (RP) regions and is considered a SLO. The WP consists of lymphocytes and mature and developing plasma cells, while the RP consists of macrophages, erythrocytes and plasma cells (213, 215). Antigen stimulation, leading to Ab synthesis, has been described in the cartilaginous fish spleen (213, 215). As previously stated, analysis of possible hematopoietic organs (kidney and/or gonads) in lobe-finned fish has yet to be completed (49). Structural analysis of the African lungfish spleen identified characteristics of a secondary lymphoid organ; the red pulp is likely the site of erythropoiesis, as well as site of plasma cell differentiation, similar to cartilaginous fish, as evidenced by mature and immature plasma cells (49). The WP appears to be involved in immune responses (49).

Both bony and cartilaginous fish have been shown to develop immunological memory (i.e., the ability to respond more rapidly and effectively to a pathogen that has been previously encountered). One of the first studies to identify immunological memory in fish was in rainbow trout where it was demonstrated that the secondary response to trinitrophenylated-keyhole limpet hemocyannin (TNP-KLH) was faster and of a larger magnitude than the primary response, as determined by ELISA (216). Several other studies in fish, including rainbow trout and turbot (*Psetta maxima*), have since shown that neutralizing Ab can be induced against a variety of viral, bacterial and parasitic pathogens and vaccines (217, 218). However, the response time of teleost IgM is much slower than in mammals, taking 3–4 weeks after immunization before specific titers are detected. Interestingly, some fish species, such as the Atlantic cod, do not appear to produce a specific antibody response upon immunization, despite high levels of serum Abs (219). This is likely due to the lack of MHC II genes and gene products in the Atlantic cod (87, 220).

Similar to teleost fish, the immune response time of IgM in cartilaginous fish is much longer than in mammals. Dooley and Flajnik completed a 3 year-long immunization study in the nurse shark (183). The results demonstrated that, following immunization, pentameric IgM, which localizes mainly in the plasma, was induced before other isotypes, but with a low-affinity interaction with antigens. The results also demonstrated that monomeric IgM, which is capable of entering tissues, appeared after pentameric IgM and was the main Ig involved in antigen-specific responses. A significant increase in antigen-specific IgNAR titers was also observed with a high specificity to antigen following immunization. It can take up to 28 months before the antigen-specific titer levels return to pre-immunization levels once the Ig response has reached a plateau (183). Memory was demonstrated for both monomeric IgM and IgNAR as re-immunization after a decrease in titer induced a quicker response than the primary immunization (183).

## AID and Affinity Maturation

Activation-induced cytidine deaminase (AID) is an enzyme that mediates somatic hypermutation (SHM) [i.e., a process that fine tunes the Ig, increasing its affinity (affinity maturation)], and mediates class switch recombination (CSR) (i.e., a process whereby the constant region of an antibody heavy chain is changed to a different isotype, thus changing its effector function) (221). AID in fish was first reported in channel catfish, and has since been reported in many other fish species (222, 223). Teleost fish AID differs from mammals in that it has a longer cytidine deaminase motif and substitutions in its carboxy-terminal region (224). Catfish and zebrafish AID have been demonstrated to mediate SHM in mouse fibroblasts (NIH3T3PI19) (225), while zebrafish AID can efficiently deaminate methylated deoxycytidines (226). In addition, the biochemical properties of AID from the sea lamprey, nurse shark, tetraodon, and coelacanth were recently characterized where it was found that these AIDs exhibit unique substrate specificities and optimal temperature tolerances while the lethargic enzymatic rate and affinity for ssDNA of AID are conserved (227). However, a search of the African lungfish mucosal lymphoid tissue transcriptome for AID found no evidence of expression using cartilaginous fish, teleost fish, or tetrapod AID sequences for comparison suggesting that the African lungfish may have lost AID expression in its genome (228). In addition, no AID was found in the African lungfish using RT-qPCR (228). However, other members of the apolipoprotein B mRNA-editing catalytic polypeptide (APOBEC) family (to which AID belongs) were found to be expressed in the African lungfish (228).

Affinity maturation is generated during immune responses in bony fish, as evidenced by the replacement of low-affinity Ab by intermediate-affinity Ab and eventually by high-affinity Ab in rainbow trout (229). The affinity maturation response in fish is much less efficient than mammals, likely due to the absence of GCs. Affinity maturation was also reported in the nurse shark, where purified monomeric IgM showed an increase in the intrinsic association constant to a ^3^H-ε-DNP-l-lysine ligand over a 20 month period (230). IgNAR also exhibits affinity maturation, as demonstrated by a correlation between somatic mutations and increased binding affinity in IgNAR clones from immune tissues of a hyperimmunized nurse shark (231). The affinity of pentameric IgM, however, does not increase during an immune response (183).

Although teleost fish express AID, they lack class switch recombination (CSR), likely due in part to the structure of the IgH gene (225). However, AID from teleost fish, specifically zebrafish, Japanese puffer and catfish can catalyze CSR *in vitro* in mammalian AID^−/−^ lymphocytes suggesting that teleost AID has the full catalytic functions capable for CSR (225, 232). Although it was once thought that cartilaginous fish were also incapable of CSR due to the cluster organization of their genes, it is now known that they can undergo an “unconventional” type of CSR among different IgM clusters and between IgW and IgM clusters (233).

## Major Histocompatibility Complex (MHC) and Antigen Presentation

A major function of B cells, as well as other pAPCs such as macrophages and dendritic cells, is to process and present antigen to activate T cells. T cells, however, will only recognize antigen fragments that are bound to MHC I or MHC II, cell surface proteins found on pAPCs. While the structure of MHC is conserved over various species, the genes encoding MHC demonstrate a high degree of polymorphism in mammals, lobe-finned fish, and ray-finned fish and cartilaginous fish, allowing different repertoires of peptides to be presented (234–236). In most teleost fish, MHC class I and II reside on different chromosomes, while in cartilaginous fish, and all other vertebrates, MHC I and II are found on the same chromosome (237–239). Interestingly, while MHC I and II are conserved in most jawed vertebrates, Gadiformes, such as the Atlantic cod, have lost the genes for MHC II and CD4, a co-receptor on T cells that interacts with MHC II (87, 88, 220, 240). The Atlantic cod does, however, contain more genes related to the MHC I component of the immune system, as well as the expansion of some TLR clades, compared to other vertebrates, which may help compensate for the missing MHC II and CD4 (87, 220).

Antigens that are to be presented by MHC I are processed via the immunoproteasome and transferred to the endoplasmic reticulum (ER) by transporter associated with antigen processing (TAP) where they associate with MHC I and are eventually transported to the cell membrane. MHC I is ubiquitously expressed in various tissues in teleost and cartilaginous fish including spleen and head kidney (239, 241, 242). In addition, β_2_ microglobulin, which is associated with MHC I, has been isolated in several teleost fish, as well as the nurse shark and sandbar shark (*Carcharhinus plumbeus*) (243–245). MHC-I related immunoproteasomes, as well as TAP genes, have also been identified in both bony and cartilaginous fish (234). While there have only been a few studies examining MHC I in lobe-finned fishes, MHC class I genes, including α1, α2, and α3, have been sequenced from blood of the African lungfish and muscle and skin of the West Indian Ocean coelacanth (*L. chalumnae*) (246, 247). Additionally, lmp1 and lmp2, catalytic subunits of the immunoproteasome, have been characterized in the African lungfish and were found to be induced in primary lung and kidney cell cultures by the synthetic dsRNA polyinosinic-polycytidylic acid [poly (I:C)] (248).

Antigens that are to be presented by MHC II are endocytosed, digested in lysosomes and loaded onto MHC II molecules prior to their migration to the cell surface. MHC II genes have been identified in teleost fish, cartilaginous fish, and the African coelacanth (173, 249, 250). Teleost MHC class II genes can be organized into three groups based on sequence features such as insertions and deletions (250). It has been shown that MHC class II affects resistance to bacterial pathogens, including *Aeromonas salmonicida* in Atlantic salmon (251). Likewise, challenge with *Vibrio harveyi* increased expression of MHC II B mRNA in the gill, liver, and spleen of the white bamboo shark (*Chiloscyllium plagiosum*), similar to teleost fish (252). The identification and characterization of MHC I and II genes in both bony and cartilaginous fish, with the exception of the Gadiformes lineage, suggests that MHC is generally well conserved in these species.

## The Adaptive Cellular Response: T Cells

T cells possess a TCR which recognizes a specific antigen and is formed using RAG-mediated V(D)J rearrangement for the development of diverse repertoires. However, unlike the BCR, the TCR is always membrane bound and only recognizes antigen when presented in the context of MHC I or II (3). T cells are classified into 2 main populations: CD8+ cytotoxic T-cells (Tc) which interact with MHC class I and CD4+ helper T cells (Th) which interact with MHC class II. In addition to MHC, all TCR possess a CD3 complex and recognize co-stimulatory (e.g., CD28) and co-inhibitory (e.g., CTLA-4) molecules. In both bony and cartilaginous fish, and similar to mammals, T cells are produced in the thymus. Research in sea bass detected T cells in the developing GALT at the same time as in the thymus, suggesting that the gut may also be a primary lymphoid organ for T cells in bony fish (33).

## T Cell Receptor

**Figure 4:**
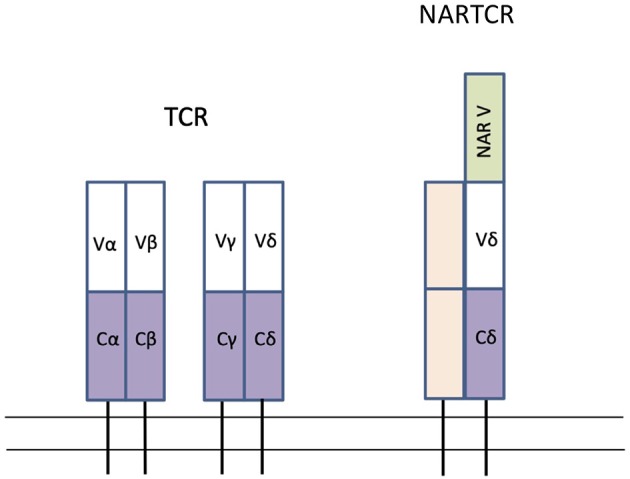
Schematic of the conventional TCR forms found in all Gnathostomes and NARTCR found in cartilaginous fish. Rectangles represent Ig super-family domains; V represent variable domains (white), C represents constant domains (purple) and NAR V represents extra variable domain in NARTCR (green). Modified from Criscitiello et al. (162), Roux et al. (253), and Criscitiello (254).

TCRs are type I transmembrane glycoproteins with extracellular V and C Ig domains and a short cytoplasmic tail (Figure 4). This structure is conserved in almost all vertebrates (255). The TCR is found in two forms: a heterodimer of α and β chains (αβ-TCR) or a heterodimer of γ and δ chains (γδ-TCR), linked by disulphide bonds. Most T cells contain the αβ-TCR, while γδ-T cells account for 1–10% of T cells in the blood of mammals, and 8–20% of total lymphocytes in various tissues of the zebrafish (256). *In situ* hybridization experiments in the nurse shark identified higher levels of TCR α and β in the central cortex of the thymus but weaker expression in the medulla and subcapsular region. Expression of TCR γ and δ were also high in central cortical cells but were most highly expressed in the subcapsular region. TCR δ was the most highly expressed TCR chain in the medulla (27).

The genes for TCR-α, β, γ, and δ are diversified using V(D)J rearrangement and have been identified in teleost fish, cartilaginous fish, and coelacanths (173, 255, 257, 258). In both bony fish and cartilaginous fish, the TCR gene segments are in the translocon arrangement, similar to mammals (27, 259). While the structure of TCR is generally well-conserved among all vertebrates, there are several unusual aspects of the cartilaginous fish TCR. Two forms of TCR-δ have been identified in cartilaginous fish, one form having an extra V domain that is closely related to IgNAR (and thus given the name NARTCR) (162, 253). It is hypothesized that the NARTCR-δ chain dimerizes with a TCR-γ chain that lacks the additional domain and therefore NARTCR-δ V domain does not have a binding partner (254). Another unique aspect of shark TCR is that some TCR-δ chains may be formed from a trans-rearrangement of Ig heavy chain V segments with D, J, and C segments of TCR-δ (162). Finally, sharks use AID and SHM to diversify the shark TCR-γ and -α chains (27, 259). SHM is not known to diversify the TCR in any other vertebrates (27, 259). Figure 4 depicts a schematic of a conventional TCR found in gnathostomes and a NARTCR found in cartilaginous fish.

## TCR Co-Receptors

The αβ subtypes can be further divided into helper CD4+ cells (Th) or cytotoxic CD8+ cells (Tc). CD4+ T cells are stimulated by peptides presented via MHC-II molecules and, when activated, CD4+ T cells release cytokines that can activate and regulate responses elicited by the antigen (260). The CD4 molecule is a single protein with four extracellular Ig-like domains and a cytoplasmic tail containing a CxC motif which interacts with the tyrosine kinase Lck, initiating intracellular signaling (261). While tetrapods contain a single CD4 molecule with four Ig domains, two types of CD4 molecules have been described in bony fish: CD4-1 containing four Ig domains and CD4-2 which contains either two or three Ig domains, depending on the species (255). In addition, salmonids contain two CD4-2 molecules (CD4-2a and CD4-2b) (262). An early study of the elephant shark genome suggested that CD4, as well as CD4 associated genes involved in the differentiation (RORC, FOXP3) and function (IL-4, IL-5, IL-13, IL-9, IL-21) of CD4+ cell lineages were missing from the genome (54). Although this study identified several CD4/Lag3-like molecules, they lacked the C-terminal intracellular CxC motif required for interacting with Lcks suggesting that CD4 is absent or not functional in the cartilaginous fish genome. However, a more recent genomic study by Redmond et al. (263) in the Small-spotted catshark used newly available sequence datasets and found putative sequences for CD4 T-cell associated genes including IL-4/IL-13, IL-21, IL-23, IL-27, IL-6Ra, IL-12R, and FOXP3, suggesting that cartilaginous fish do in fact have CD4 T-cell subsets, although more work is still required to fully understand the T cell subsets present in cartilaginous fish, as well as their biological roles (263).

CD8+ T cells are activated by peptides presented via MHC-I molecules and secrete cytotoxins such as perforin and granzymes that initiate apoptosis in the target cells. The CD8 molecule can be in one of two forms: a homodimer formed from two α-chains (CD8αα) or a heterodimer formed from one α- and one β- chain (CD8αβ) (264). Both CD8 chains have been characterized in multiple teleost fish and cartilaginous fish (27, 54, 265, 266). Teleost and cartilaginous fish CD8 exhibit an extracellular Ig-like domain, but the domain has a CxH motif in the cytoplasmic tail, instead of the CxC motif found in mammals, suggesting that CxH represents a primordial Lck binding site (162, 267).

T-cell activation is triggered via antigen:MHC recognition by the TCR and mediated via CD3. All TCR have a short cytoplasmic tail and therefore need to partner with CD3, a complex of transmembrane proteins with intracellular domains containing the conserved motif known as immunoreceptor tyrosine-based activation motif (ITAM). Characterization studies of CD3 in teleost fish have identified a conserved structure of CD3 between teleost fish and mammals (268, 269). Genes encoding the CD3 chains have been annotated in the elephant shark genome and were recently cloned in the small-spotted catshark (*S. canicula*) where two copies of CD3 were observed (54, 270). Three CD3 chains have also been identified in the coelacanth genome (173). The sequence homology of all 3 chains encoded in the coelacanth genome were distinct from other fishes but grouped together with the corresponding molecules found in avians and mammals (173).

The initial interaction of TCR/MHC/peptide is not sufficient to fully induce activation of naïve T cells and therefore T cells require additional co-stimulatory signals. This is provided by the interactions between CD28, a co-stimulatory factor expressed on T cells, and B7.1 (CD80) and B7.2 (CD86) ligands on the APC. In contrast, binding of B7.1 and B7.2 to CTLA4, a powerful negative regulator of T cell activation, exerts an inhibitory effect on T cell activation. Both CD28 and CLTA4, as well as orthologs of B7.1 and B7.2, have been identified in several teleost species (271–273). The binding sites for B7.1 and B7.2 are conserved in teleost fish CD28 and CTLA4, indicating that CD28 and CTLA4 recognize a B7-like receptor (271). In addition, viral infection in rainbow trout increases CTLA-4 expression, while CD28 remains constitutively expressed, similar to mammals, suggesting that these molecules may have similar roles as their mammalian orthologs (271). Putative CD28, CLA-4, and B7 genes have been annotated in the elephant shark genome, while CD28 has been identified in the coelacanth genome, however the function of these co-receptors in many fish species remains to be fully investigated (54, 173).

## The T-Cell Effector Response

Upon activation of CD4+ cells, naïve cells can differentiate into specific subsets including Th1, Th2, Th17, and inducible T-regulatory (Treg) cells; each subset defined by their cytokine production (274). Activation of CD8+ cells induces differentiation into cytotoxic effector cells which release cytotoxins that induce apoptosis of the target cell.

## CD4+ Th Cells

The structures of several orthologs and paralogs of Th cytokines, as well as their functions, have been characterized in both teleost fish and cartilaginous fish and are reviewed in Secombes et al. (275, 276) and Secombes and Wang (277). In brief, two forms of IFNγ, produced by Th1 cells, IFNγ, and IFNγ rel, have been identified in teleost fish including Atlantic salmon, rainbow trout, and ginbuna crucian carp (*Carassius carassius*), while one form has been identified in fugu (278–280). Recombinant IFNγ (r-IFNγ) was found to increase the expression of anti-viral and inflammation-relevant genes, as well as increase ROS and NO production in zebrafish, rainbow trout and goldfish macrophages, indicating a similar function as mammalian IFNγ (281, 282). A single of copy IFNγ has been identified in the Elephant shark genome (54). Three Il-4/13 genes (IL-4/13A, IL-4/13B1, and IL-4/13B2), produced by Th cells, have been characterized in salmonids (276, 283, 284). Intraperitoneal injection of r-IL-4/13A in zebrafish increased the number of IgZ+ B cells circulating in the blood, compared to a PBS control injection (285), while r-IL-4/13A in rainbow trout modulates the expression of a number of Th2 genes (286). While Venkatesh et al. (54) found no IL-4/13 genes in the elephant shark genome, subsequent interrogation of the genome by Dijkstra (287) found three putative IL-4/13 genes. In addition, Redmond et al. identified a IL-4/IL-13 gene in the small spotted catshark genome (263). Analysis of the coelacanth genome failed to identify Il-4 (173). The IL-17 family in teleost fish, produced by Th17 cells, has several members (A-F) which are structurally related to orthologous proteins in mammals (288, 289). Two homologs of the IL-17 family, IL-17B and IL-17D have been identified in teleost fish, as well as several isoforms of molecules termed IL-17A/F1-3, IL-17C, and IL-17E (290). r-IL-17A/F2 induced the expression of antibacterial peptide β-defensin-3 and the pro-inflammatory cytokines IL-6 and IL-8 in rainbow trout splenocytes, suggesting its role in antibacterial defenses (289). Several IL-17 family members have been found in a cartilaginous fish genome (*C. milii*) including IL-17A/F, IL-17B, IL-17C, and IL-17D (275). One copy of the IL-10 gene, produced by Treg cells, is found in most species of teleost fish, although two copies have been identified in rainbow trout and European common carp (291, 292). Sequences with homology to IL-10 were found in the spiny dogfish (*S. acanthias*), elephant shark, and coelacanth genomes (173, 275). These studies, among many other fish cytokine studies, indicate that the structure of cytokines released from Th cells is relatively conserved between ray-finned fish, lobe-finned fish, and cartilaginous fish.

## CD8+ Cytotoxic T Cells

Cytotoxic T cells kill their targets via two mechanisms: the secretory and non-secretory pathways, both of which induce apoptosis. The secretory pathway releases granular toxins such as perforin and serine proteases called granzymes which work together to induce apoptosis (293). The non-secretory pathway involves the engagement of target-cell death receptors, such as Fas, located on the cell surface of the cytotoxic T cells, which results in caspase-dependent apoptosis (294).

The secretory pathway has been identified in many different fish species. A perforin-like molecule has been characterized in several teleost species (295, 296). The killing function of α/β TCR alloantigen specific cytotoxic clones was inhibited in channel catfish by treatment with concanamycin A, a perforin inhibitor (297). Similarly, treatment of ginbuna crucian carp CD8α+ lymphocytes with concanamycin A partially inhibited their function in a dose dependent manner, suggesting that the perforin-mediated pathway in teleost fish is similar to that of higher vertebrates (298). Granzyme has also been recently identified in ginbuna crucian carp (gcGzm) and has a similar primary structure to that of mammals (299). Expression of gcGzm mRNA was greatly enhanced by allo-sensitization and infection with *Edwardsiella tarda*, indicating that gcGzm is involved in cell mediated immunity (299). In spite of the absence of CD4 and associated CD4 genes, many cytotoxic T cell related genes, including perforin and granzyme, have been identified in the elephant shark genome, suggesting that such cell types are present in cartilaginous fish (54).

While the non-secretory pathway has not been as thoroughly studied in fish as the secretory pathway, the FasL protein has been identified in channel catfish, tilapia (*O. niloticus*), and gilthead sea bream (300–302). Recombinant FasL protein from Japanese flounder (*Paralichthys olivaceus*) induced apoptosis in a flounder cell line, indicating that fish possess a similar Fas ligand system (303). FasL has yet to be identified in cartilaginous and lobe-finned fish.

## Conclusion and Future Directions

Comparative studies in fish help to reveal the evolutionary history of the immune system. Whereas, innate immunity is present in all multicellular organisms, an adaptive immune system, based on VLRs, B-like, and T-like cells is found in jawless vertebrate, while an adaptive immune system, based on an Ig/TCR/MHC system, evolved with the appearance of jawed vertebrates. Research on the fish immune systems is continuously on the rise, however there is still much to be discovered. For example, there is limited information on TLR ligands, especially in cartilaginous and lobe-finned fish, as well as limited information on complement proteins in lobe-finned fish. In order to gain a better understanding of the lobe-finned fish immune system, the site(s) of hematopoiesis must be determined. There is also limited knowledge, compared to mammals, on the homing and recirculation of B and T cells in ray-finned fish, cartilaginous fish, and lobe-finned fish. Much of this knowledge will only be gained with the development of the appropriate reagents and techniques. The development of cell lines for cartilaginous and lobe-finned fish will aid in determining basic cell biology, one of the first steps in understanding the immune system. Many comparative fish immunology studies are genome-based, and fish genomes are often not well-assembled and/or annotated. The development of high-quality, well-assembled, and annotated genomes in fish species will allow the identification of more immune-relevant transcripts, such as NITRs. In addition, the lack of protein-specific antibodies for fish is hindering many research avenues, such as flow cytometry and cell-specific analyses. A comprehensive understanding of the evolution of the immune system will continue to develop as more comparative research on cartilaginous fish, lobed-finned fish, and ray-fined fish is completed.

## Author Contributions

NS reviewed the literature, generated the figures, and wrote the manuscript. SC and MR revised and edited the manuscript.

### Conflict of Interest

The authors declare that the research was conducted in the absence of any commercial or financial relationships that could be construed as a potential conflict of interest.
